# Erythropoietin-Derived Nonerythropoietic Peptide Ameliorates Experimental Autoimmune Neuritis by Inflammation Suppression and Tissue Protection

**DOI:** 10.1371/journal.pone.0090942

**Published:** 2014-03-06

**Authors:** Yuqi Liu, Bangwei Luo, Fuyu Han, Xiaoming Li, Jian Xiong, Man Jiang, Xioafeng Yang, Yuzhang Wu, Zhiren Zhang

**Affiliations:** Institute of Immunology, Third Military Medical University of People’s Liberation Army, Chongqing, China; Innsbruck Medical University, Austria

## Abstract

Experimental autoimmune neuritis (EAN) is an autoantigen-specific T-cell-mediated disease model for human demyelinating inflammatory disease of the peripheral nervous system. Erythropoietin (EPO) has been known to promote EAN recovery but its haematopoiesis stimulating effects may limit its clinic application. Here we investigated the effects and potential mechanisms of an EPO-derived nonerythropoietic peptide, ARA 290, in EAN. Exogenous ARA 290 intervention greatly improved EAN recovery, improved nerve regeneration and remyelination, and suppressed nerve inflammation. Furthermore, haematopoiesis was not induced by ARA 290 during EAN treatment. ARA 290 intervention suppressed lymphocyte proliferation and altered helper T cell differentiation by inducing increase of Foxp3^+^/CD4^+^ regulatory T cells and IL-4^+^/CD4^+^ Th2 cells and decrease of IFN-γ^+^/CD4^+^ Th1 cells in EAN. In addition, ARA 290 inhibited inflammatory macrophage activation and promoted its phagocytic activity. In vitro, ARA 290 was shown to promote Schwann cell proliferation and inhibit its inflammatory activation. In summary, our data demonstrated that ARA 290 could effectively suppress EAN by attenuating inflammation and exerting direct cell protection, indicating that ARA 290 could be a potent candidate for treatment of autoimmune neuropathies.

## Introduction

Guillain-Barré Syndrome (GBS), which is characterized by motor disorders such as weakness or paralysis, as well as variable sensory disturbances, is the world’s leading cause of acute autoimmune neuromuscular paralysis and caused by an autoimmune attack on the peripheral nervous system. Existing treatments of inflammatory polyneuropathies can be divided into supportive management, such as good intensive care and respiratory assistance, as well as active treatment including plasma exchange and intravenous immunoglobulin [1]. Nevertheless, only around 65% patients with GBS respond to plasma exchange or intravenous immunoglobulin, about 8% of GBS patients die, and up to 20% remain disabled despite modern treatment [2]. Even in those who recover well, residual weakness and loss of motor units can usually be detected and could explain that the fatigue is a common problem [3]. Therefore, more efficacious therapeutic options represent an urgent medical need for polyneuropathies.

Experimental autoimmune neuritis (EAN) is a helper T cell-mediated inflammatory demyelinating disease of the peripheral nervous system (PNS) that mirrors many clinical and immunological features of the human acute inflammatory demyelinating polyradiculoneuropathies (AIDP), a subtype of GBS [4], [5]. Pathologically, EAN is characterized by breakdown of the blood-nerve barrier, robust accumulation of reactive T cells and macrophages and demyelination in the PNS [5]. Further Schwann cells are the myelinating glial cells of the PNS that support and ensheath axons with myelin to enable rapid saltatory signal propagation in the axon. Schwann cells can also modulate local immune responses by recognizing and presenting antigens and may influence and terminate nerve inflammation by secreting cytokines in EAN [6]. Therefore, Schwann cells, reactive lymphocytes and macrophages orchestrate a robust local inflammation that are essential for the development and recovery of EAN.

Erythropoietin (EPO) is a pleiotropic cytokine that was initially identified as an essential regulator of red blood cell production through the homodimer EPO receptor (EPOR_2_) expressed on the surface of hematopoietic progenitor cells and is widely applied to treat the anemia of various origins [7]. However, recently EPOR expression and biologic response to EPO have been observed in a variety of other cells, such as Schwann cells, endothelial cells, neuron, cardiac cells and different immune cells and accumulated studies have shown that EPO signaling contributes to wound healing, angiogenesis and the body’s innate response, indicating that EPO has cyto-protective and anti-inflammatory effects as well [8]–[13]. And the tissue-protective effects of EPO is considered to induced via activation of the EPOR-CD131 complex, whose affinity for EPO is 100 times lower than that of the homodimer EPOR_2_ [14]. Although EPO is accepted as a safe therapeutic for treating anaemia, the clinical use of EPO as a cytoprotective drug raises concerns of its possible adverse side effects, such as thromboembolism and hypertension [15]–[17] because the tissue protective doses of EPO are much higher than those needed for stimulation of haematopoiesis [11]. Therefore, EPO analogues that retain their tissue-protective properties but lack erythropoietic activity have been developed.

In EAN, EPO treatment reduces the clinical score, suppresses the inflammation, reduces the demyelination and protects from axonal loss, suggesting that EPO could be a potent candidate for treatment of immune neuropathies [18]–[20]. However, a significant proportion of GBS patients have some degree of failure of axon regeneration and target reinnervation after the acute phase, indicating that a relative long-term application of EPO is necessary for GBS treatment. So EPO analogues having tissue-protective properties without stimulating hematopoiesis might be a better option for GBS treating.

ARA 290 is a recently developed nonerythropoietic EPO derivatives, which selectively binds the heteromeric EPOR-CD131 complex. ARA 290 is an 11 amino acid peptide that mimics a portion of helix B of the EPO molecule [21]. Herein, effects together with potential mechanisms of ARA 290 in EAN recovery were studied.

## Materials and Methods

### 1 Animals

Male Lewis rats (8–10 weeks, 200–220 g, Vital River, Beijing, China) were housed under a 12 h light and 12 h dark cycle with free access to food and water. All animal experimental procedures in this study were approved by the Animal Care and Use Committees of Third Military Medical University (approval number: TMMU 12-07-12). All efforts were made to minimize the number of animals and their suffering.

### 2 EAN Induction and Intervention

Lewis rats were immunized by subcutaneous injection into both hind footpads with 100 µL of an inoculum containing 100 µg of synthetic neuritogenic P2 57–81 peptide (Chinapeptide Corporation, Shanghai, China). The peptide was dissolved in phosphate buffered saline (PBS) (2 mg/mL) and then emulsified with an equal volume of complete Freund’s adjuvant (Sigma, St Louis, MO, USA) containing 4 mg/mL mycobacterium tuberculosis to get a final concentration of 2 mg/mL.

After immunization, rats were monitored daily for body weight and neurological signs of EAN, which were scored as follows: 0 = normal, 1 = reduced tonus of tail, 2 = limp tail, impaired righting, 3 = absent righting, 4 = gait ataxia, 5 = mild paresis of the hind limbs, 6 = moderate paraparesis, 7 = severe paraparesis or paraplegia of the hind limbs, 8 = tetraparesis, 9 = moribund, 10 = death.

For intervention, EAN rats received intraperitoneal (i.p.) injection of ARA290 (Chinapeptide Corporation, Shanghai, China) daily from Day 7 to Day 21. The ARA290 was dissolved in PBS and the same volume of PBS was given to control group.

### 3 Immunohistochemistry and Electron Microscopy

To evaluate inflammatory cell infiltration and pathological changes in the PNS, six ARA 290-treated or control EAN rats were sacrificed at Day 14 or Day 21. Rats were deeply anesthetized with ether and perfused intracardially with 4°C, 4% paraformaldehyde in PBS. Left and right sciatic nerves were quickly removed and post-fixed in 4% paraformaldehyde overnight at 4°C. Tissues were cut into two equally long segments, embedded in paraffin, serially sectioned (3 µm) and mounted on silan-covered slides.

After dewaxing, cross-sections were boiled (in a 600 W microwave oven) for 15 min in citrate buffer (2.1 g sodium citrate/L, pH 6). Endogenous peroxidase was inhibited with 1% H_2_O_2_ in methanol for 15 minutes. Sections were incubated with 10% normal pig serum (Biochrom, Berlin, Germany) to block non-specific binding of immunoglobulin and then with the CD68 antibody for macrophages (1∶100; Serotec, Oxford, UK) or CD3 antibody for T lymphocytes (1∶50; Serotec, Oxford, UK). Antibody binding to tissue sections was visualized with a biotinylated IgG F(ab)_2_ secondary antibody fragment (DAKO, Hamburg, Germany). Subsequently, sections were incubated with a horseradish peroxidase-conjugated streptavidin complex (DAKO, Hamburg, Germany), followed by development with diaminobenzidine (DAB) substrate (Fluka, Neu-Ulm, Germany). Finally, sections were counterstained with Maier’s Hemalum.

To evaluate immunostaining data, the percentages of areas of immunoreactivity (IR) to areas of sciatic nerve cross-sections were calculated. Briefly, images of sciatic nerve cross-sections were captured under 50× magnification using Nikon Coolscope (Nikon, Düsseldorf, Germany) with fixed parameters. Images were analysed using MetaMorph Offline 7.1 (Molecular Devices, Toronto, Canada). Areas of IR were selected by colour threshold segmentation and all parameters were fixed for all images. Areas of sciatic nerve cross-sections were manually selected and were measured using software MetaMorph Offline 7.1. For each EAN rat, four cross-sections from root and middle levels of both sides were analyzed. Results were given as arithmetic means of percentages of areas of IR to areas of sciatic nerve cross-sections and standard errors of means (SEM).

The routine HE staining was applied to show mononuclear cell infiltration. Histological changes between different groups were compared by an established semi-quantitative method [22]. Briefly, four cross-sections from root and middle level of both sides of sciatic nerves from EAN rats were analyzed. All perivascular areas present in cross-sections were evaluated by two observers unaware of intervention, and the degree of pathological alteration was graded semi quantitatively on the following scale: 0 = normal perivascular area; 1 = mild cellular infiltrate adjacent to the vessel; 2 = cellular infiltration plus demyelination in immediate proximity to the vessel; 3 = cellular infiltration and demyelination throughout the section. Results were given as mean histological score. All slides were blindly scored by two independent researchers.

To evaluate EAN sciatic nerve axon degeneration/regeneration and demyelination/remyelination following ARA 290 intervention, electron microscopy was applied. Rats were deeply anesthetized by ether, perfused with PBS and fixed with 4°C, 4% paraformaldehyde in PBS, then quickly fixed in 2.5% glutaraldehyde at 4°C for 24 h. The block was further cut into sagittal fragment and post-fixed in 1% osmium tetroxide for 2 h. After dehydration with cold acetone, the fragment was embedded in araldite epoxy resin, cut sagittally in half and stained with toluidine blue. The fragment was cut into 60-nm ultra-thin sections, and then stained with uranyl acetate and lead citrate. All images were obtained with a transmission electron microscope (TECNAI10, Philips) at 80 kV. Furthermore, the axon degeneration and demyelination was semi-quantified by a reported method [19].

### 4 Tissue Preparation, RNA Isolation, Reverse Transcription and Real-time PCR

Following intervention, EAN rats were perfused intracardially with 4°C PBS under anaesthesia and then sciatic nerves and inguinal lymph nodes were quickly removed and stored in liquid nitrogen until RNA isolation. Total RNA was isolated using Trizol LS Reagent (Invitrogen, Carlsbad, CA, USA) and reverse transcribed into cDNA using Quantscript RT Kit (TIANGEN Biotech, Beijing, China).

The cDNA was used to measure the relative expression of genes using RealMasterMix (SYBR green I) according to the manufacturer’s protocol (TIANGEN Biotech, Beijing, China). Real-time measurements of gene expression were performed with the DNA Engine Opticon 2 Real-Time Cycler PCR detection system (Bio-Rad Lab., Richmond, CA, USA). Primers used to measure gene expression are: interleukin-1β (IL-1β, sense, TGC TGA TGT ACC AGT TGG GG; antisense, CTC CAT GAG CTT TGT ACA AG), IL-6 (sense, GCC CTT CAG GAA CAG CTA TG; antisense, CAG AAT TGC CAT TGC ACA AC), interferon-γ (IFN-γ, sense, AAA GAC AAC CAG GCC ATC AG; antisense, CTT TTC CGC TTC CTT AGG CT), IL-4 (sense, TGA TGG GTC TCA GCC CCC ACC TTG C; antisense, CTT TCA GTG TTG TGA GCG TGG ACT C), IL-17 (sense, TGG ACT CTG AGC CGC ATT GA; antisense, GAC GCA TGG CGG ACA ATA GA), transforming growth factor-β (TGF-β, sense, TGA ACC AAG GAG ACG GAA TAC AGG; antisense, TAC TGT GTG TCC AGG CTC CAA ATG), IL-12p35 (sense, TGA AGA CCA CGG ACG ACA; antisense, TGT GAT TCA GAG ACC GCA TTA G), Foxp3 (sense, GCA CAA GTG CTT TGT GCG AGT; antisense, TGT CTG TGG TTG CAG ACG TTG T), IL-10 (sense, CCT GCT CTT ACT GGC TGG AG; antisense, TCT CCC AGG GAA TTC AAA TG), RORγt (sense, CGC ACC AAC CTC TTC TCA CG; antisense, GAC TTC CAT TGC TCC TGC TTT C), GATA-3 (sense, CTC TCC TTT GCT CAC CTT TTC; AAG AGA TGC GGA CT GGA GTG) and β-actin (sense, CCG TCT TCC CCT CCA TCG T; antisense, ATC GTC CCA GTT GGT TAC AAT GC).

### 5 Flow Cytometric Analysis of Foxp3^+^ Regulatory T cells and Th1/Th2 Cells in Spleen

T regulatory cells were identified as Foxp3^+^/CD4^+^ cells and Th1/Th2 cells were identified as IFN-γ^+^/CD4^+^ cells and IL-4^+^/CD4^+^ cells. In short, rat spleens were dissected under sterile conditions and passed through a 40 mm cell strainer followed by Red Blood Cell Lysis Buffer (Biolegend, San Diego, USA). For determination of Th1/Th2 cells, splenocytes were incubated for 6 hours in the presence of 50 ng/mL phorbol-12-myristate-13-acetate (Sigma, St Louis, MO, USA), 1 mg/mL ionomycin (Sigma, St Louis, MO, USA) and 1 µg/mL Brefeldin A (Sigma, St Louis, MO, USA) in flat bottom 96-well plates in RPMI 1640 media (Gibco, Grand Island, NY) containing penicillin (100 U/mL), streptomycin (100 U/mL) and 10% fetal calf serum at a density of 10^6^ cells/mL at 37°C and 5% CO_2._ Thereafter splenocytes were fixed and permeabilized. AlexaFlour488-labelled Foxp3 antibody (Biolegend, San Diego, USA) and PE-labelled CD4 antibody (Biolegend, San Diego, USA) were used for staining of Foxp3^+^/CD4^+^ cells, and FITC-labelled IFN-γ antibody and PE-labelled CD4 antibody were used for staining of IFN-γ^+^/CD4^+^ cells and PE-labelled IL-4 antibody (Biolegend, San Diego, USA) and APC-labelled CD4 antibody (Sungene Biotech, Tianjin, China) were used for staining of IL-4^+^/CD4^+^ cells according to the standard protocol. For all staining, isotype controls were used. Following staining, cells were washed and suspended in PBS, and then analyzed by a FACScan (BD Biosciences, Franklin Lakes, NJ, USA). Mononuclear cells were gated by forward and sideward scatter.

### 6 RSC96 Schwann Cell Culture and Intervention

Rat RSC96 Schwann cells were grown in complete Dulbecco’s modified eagle medium (DMEM) media (Gibco, Grand Island, NY) containing penicillin (100 U/mL), streptomycin (100 U/mL) and 10% fetal calf serum at 37°C and 5% CO_2_.

To analyse the effects of ARA 290 on inflammatory cytokine expression by RSC96 Schwann cells, 10^6^ cells were seeded into 6-well cell culture plates in triplicates and cultured overnight. Afterwards cells were washed twice by PBS, and then ARA 290 of different doses were added into the culture with or without 1 µg/ml lipopolysaccharide (LPS) for overnight. Thereafter, mRNA levels of inflammatory cytokines were measured using following primers: TNF-α (sense, TGA TCG GTC CCA ACA AGG A; antisense, TGC TTG GTG GTT TGC TAC GA), iNOS (sense, TCT GTG CCT TTG CTC ATG ACA; antisense, TGC TTC GAA CAT CGA ACG TC) and β-actin (sense, CCG TCT TCC CCT CCA TCG T; antisense, ATC GTC CCA GTT GGT TAC AAT GC).

In Schwann cell proliferation assays, cells were cultured in 1% fetal calf serum and treated with different doses of ARA 290 for 24 h and compared to proliferation of cells in normal 10% fetal calf serum. Cell proliferation was determined by Cell Counting Kit-8 Assay (Dojindo, Kumamoto, Japan) according to the manufacturer’s protocol.

### 7 Macrophage RAW 264.7 Culture and Intervention

The immortalized murine macrophage cell line RAW 264.7 was grown in complete Dulbecco’s modified eagle medium (DMEM) media (Gibco, Grand Island, NY) containing penicillin (100 U/mL), streptomycin (100 U/mL) and 10% fetal calf serum at 37°C and 5% CO_2_.

To determine effects of ARA 290 on inflammatory cytokine expression, 10^6^ RAW 264.7 cells were seeded into 6-well cell culture plates in triplicates and cultured overnight. Afterwards, cells were washed twice with PBS, and then different doses of ARA 290 were added to culture with or without 1 µg/ml LPS overnight or for 24 hours. Thereafter, mRNA levels of inflammatory cytokine was measured using following primers: β-actin (sense, TGG AAT CCT GTG GCA TCC ATG AAA; antisense, TAA AAC GCA GCT CAG TAA CAG TCC G), TNF-α (sense, AAC TAG TGG TGC CAG CCG AT; antisense, CTT CAC AGA GCA ATG ACT CC).

For phagocytosis assay, FITC-conjugated dextran (FITC-dextran) was used. Briefly, 0.5 mg/ml FITC-dextran (mw. 40,000, Sigma, St Louis, MO, USA) was incubated with Raw264.7 cells for 30 min at 37°C; uptake was stopped by washing the cells with ice-cold FACS buffer. Control for dextran internalization was established by incubating cells with FITC-dextran on ice. Data were acquired by a FACScan (BD Biosciences, Franklin Lakes, NJ, USA).

### 8 Mixed Lymphocyte Cell Proliferation Assay

Spleen mononuclear cells (MNC) were prepared as described above. 200 µl aliquots of MNC suspensions were cultured in triplicates in round-bottomed 96-well polystyrene microtiter plates (Nunc, Copenhagen, Denmark) at a cell density of 10^6^ cell/ml. For lymphocyte stimulation, Con A was added to cultures at a final concentration of 20 µg/ml. For specific lymphocyte stimulation, P2 peptide 57–81 were added to cultures at a final concentration of 10 µg/ml. Triplicate wells without Con A or specific antigen served as background controls. ARA 290 was added during the culture period with increasing concentration. After 60 hours of incubation, cells were pulsed with [^3^H]-methylthymidine (1 mCi/well) and cultured for an additional 12 hr. [^3^H]-methylthymidine incorporation was measured and the results were expressed as counts per minute (cpm).

### 9 Data Evaluation and Statistical Analysis

The unpaired t-test or Wilcoxon-Mann-Whitney test was performed to compare difference between ARA290 and control EAN rats (Graph Pad Prism 5.0 for windows). The Wilcoxon-Mann-Whitney was used to test for statistically significant differences in clinical score and weight values. Student’s t-test for unrelated samples was used to test for statistically significant differences in all other analyses. For all statistical analyses, significance levels were set at *P*<0.05.

## Results

### 1 ARA 290 Intervention Favored the Alleviation of EAN without Stimulating Hematopoiesis

**Figure 1:**
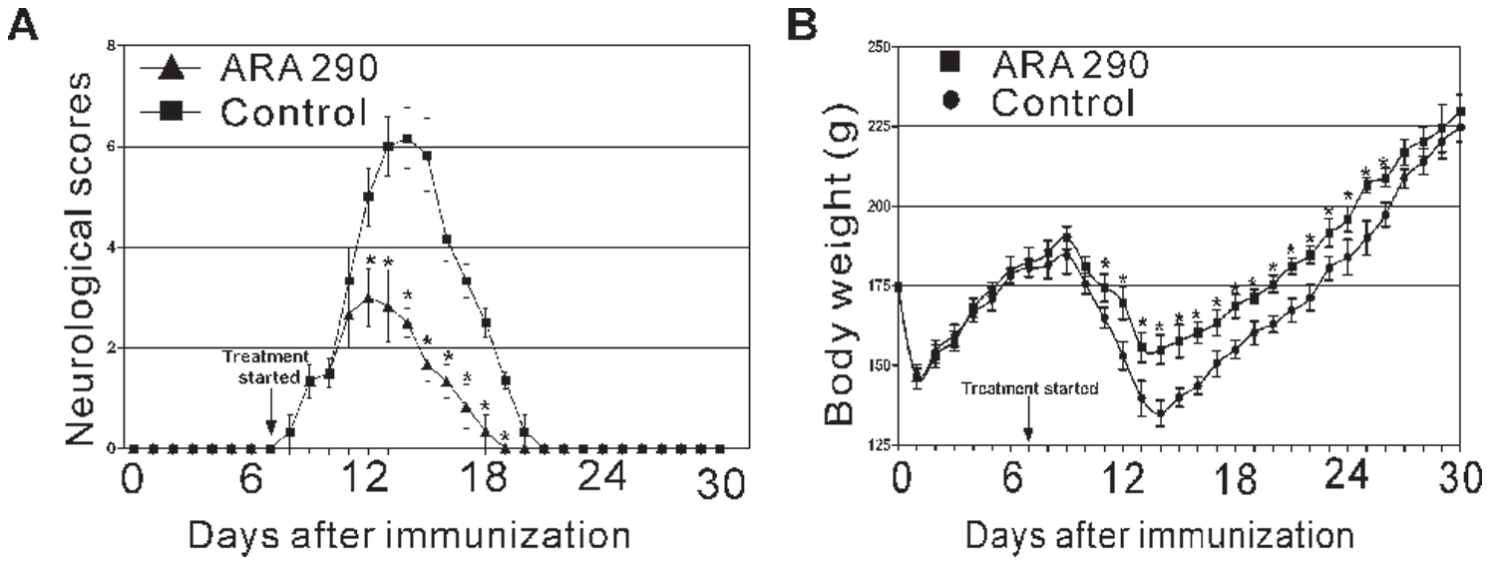
ARA 290 intervention effectively attenuated EAN disease severity. PBS or ARA 290 (30 mg/kg/day) was given to EAN rats (n = 6) from Day 7 to Day 21. All rats were monitored daily for body weight and neurological signs of EAN. **A:** ARA 290 intervention greatly decreased neurologic severity of EAN, shortened EAN duration in comparison to the PBS-treated group. **B:** ARA 290 intervention reduced body weight loss in comparison to the PBS-treated group. *: p<0.05 compared to their respective vehicle control.

EAN rats administrated ARA 290 (30 mg/kg/day) or PBS (control group) from Day 7 to 21. For PBS injection, the first neurologic sign of EAN was observed on Day 8 (0.4±0.14), the neurologic severity of EAN reached maximum on Day 14 (6.10±0.22) and disappeared around Day 21. Intervention with ARA 290 greatly decreased neurologic severity and shortened recovery time and total duration of EAN (Figure 1A). A further feature of EAN is progressive weight loss. In control and ARA 290-treated EAN rats, a rapid weight loss was observed immediately following immunization (Day 1), which was followed by slow weight gain. Control EAN rats showed weight loss during the period of neurologic disease from Day 9 to 14 post immunization, followed by rapid weight gain (Figure 1B). For ARA 290-treated EAN rats, less severe weight loss was observed compared to control (Figure 1B), indicating a much less severe disease course.

**Figure 2:**
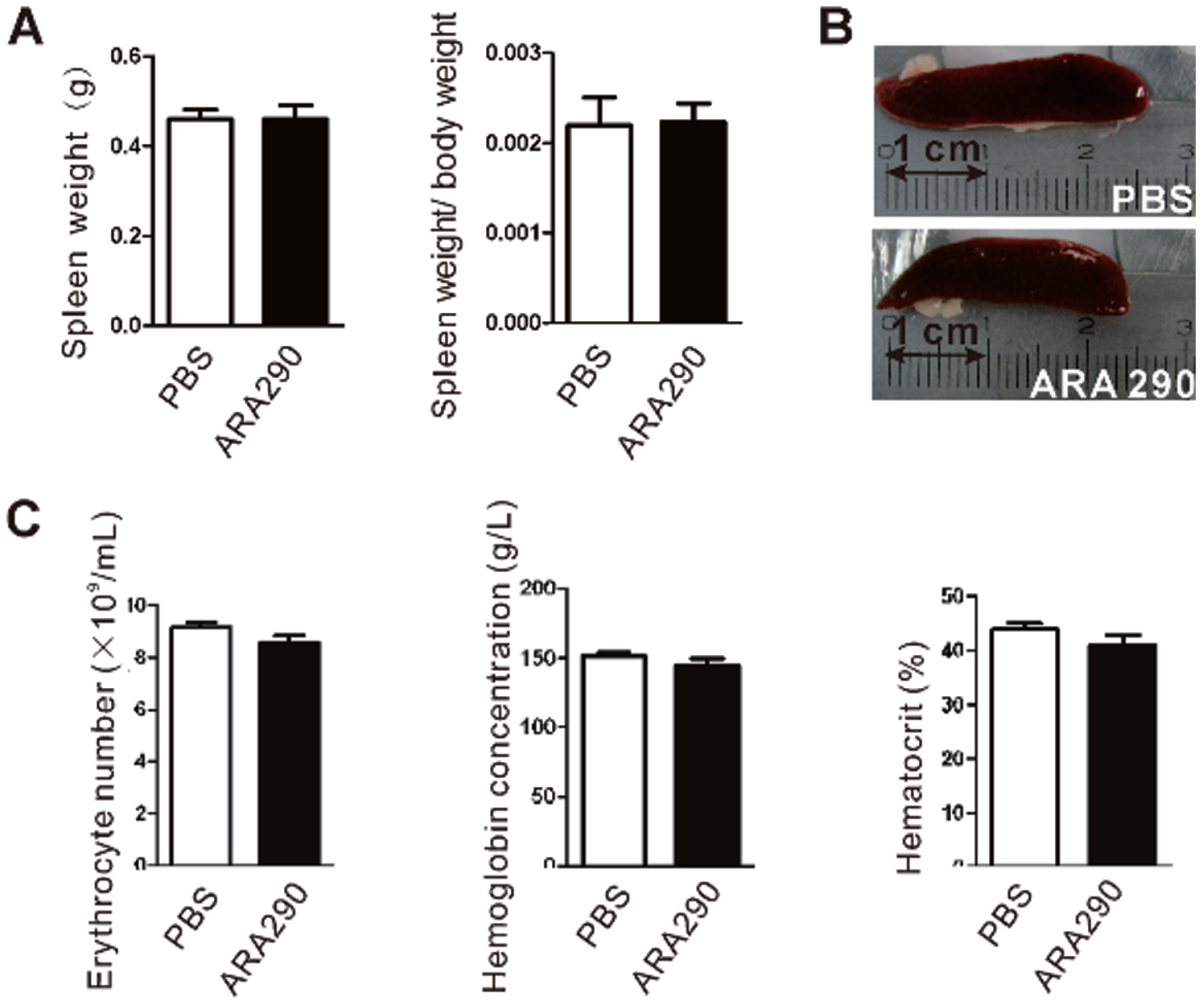
ARA 290 is not erythropoietic *in vivo*. PBS or ARA 290 (30 mg/kg/day) was given to EAN rats (n = 6) from Day 7 to Day 21 and on Day 21, rats were sacrificed to take spleen and pripheral blood for detection of erythropoietic activation. **A–B:** Representative images of spleen was recorded. ARA 290 intervention did not alter the spleen weight of treated rats compared to control. **C:** Anticoagulated blood was measured for erythrocyte cell number, hemoglobin concentration and hematocrit. ARA 290 intervention did not increase erythrocyte cell number, hemoglobin concentration or hematocrit compared to PBS group.

We next investigated whether ARA 290 also possesses hematopoietic properties during EAN intervention. Administration of ARA 290 to EAN rats once daily from Day 7 to 21 post immunization did not alter the weight of treated rats compared to control (Figures 2A, 2B). Furthermore, the numbers of peripheral red blood cells, the level of haemoglobin and the percentage of hematocrit were not changed during the injection period compared to control (Figure 2C), suggesting that ARA290 did not stimulate haematopoiesis during EAN intervention.

### 2 ARA 290 Reduced Inflammation in Peripheral Nerves of EAN Rats

**Figure 3:**
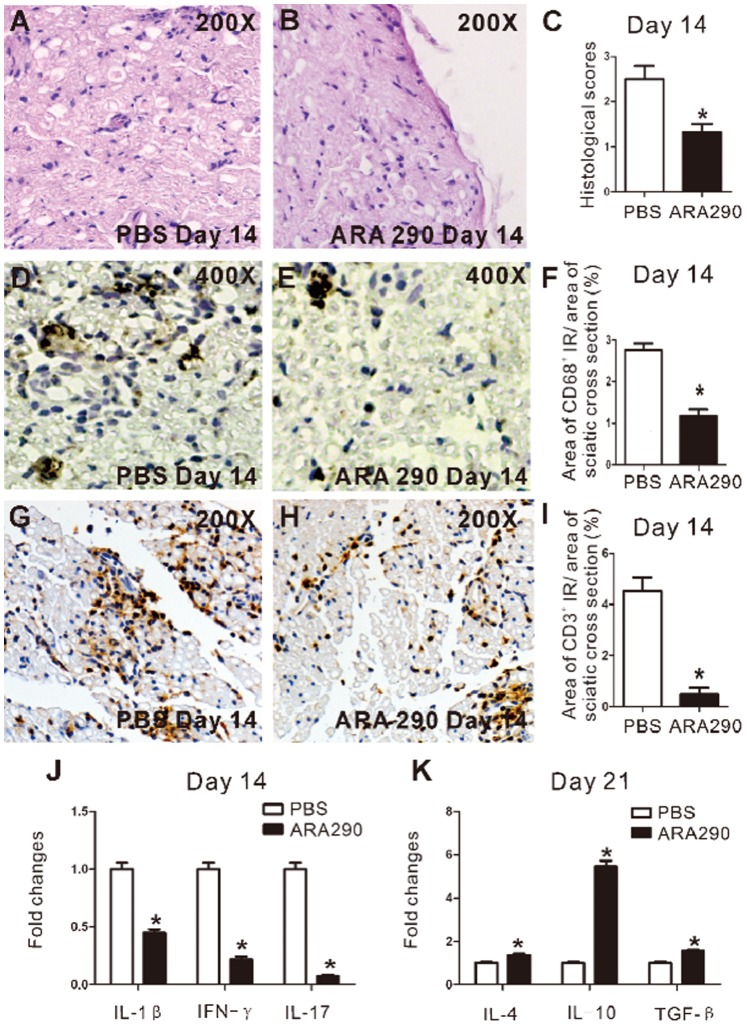
ARA 290 intervention reduced inflammatory cell infiltration and altered inflammatory cytokines expression in sciatic nerves. PBS or ARA 290 (30 mg/kg/day) was given to EAN rats from Day 7 to Day 21 and on Day 14 or Day 21, rats were sacrificed to take sciatic nerves for HE/immunohistochemical staining or RT-PCR analysis. A–C: HE staining was applied to show inflammatory infiltration of Day 14 in EAN sciatic nerves. Representative microimages showed that inflammatory cell infiltration was significantly suppressed by ARA 290 intervention compared to PBS control (n = 3). D–I: CD68 and CD3 staining was applied to show macrophages and T lymphocytes infiltration of Day 14 in EAN sciatic nerves. Representative microimages showed that macrophages (CD68^+^) and T lymphocytes infiltration was significantly suppressed by ARA 290 intervention compared to PBS control (n = 3). J–K: Sciatic nerves from Day 14 and Day 21 ARA 290-treated EAN rats were taken for RT-PCR analysis (n = 3). IL-1β, IFN-γ, IL-17 levels were reduced, while IL-4, IL-10, TGF-βlevels were increased. *: p<0.05 compared to their respective vehicle control.

EAN rats were treated by ARA 290 or PBS as described and sacrificed on Day 14 to take sciatic nerves for histological analysis (n = 4). HE staining was used to show cell infiltration. As shown in Figure 3A, obvious perivascular inflammatory cell infiltration was seen in sciatic nerves of control EAN rats. ARA 290 significantly decreased the incidence of perivascular inflammatory cell infiltration (Figure 3B). Histological changes between ARA 290-treated and PBS-treated EAN rats were further compared by an established semi-quantitative method. In sciatic nerves, the mean histological scores were markedly lower in ARA 290 group (1.47±0.05) than control group (2.5±0.08) (Figure 3C).

The attenuation of macrophage and T lymphocytes infiltration in sciatic nerves of Day 14 EAN rats following ARA 290 intervention was further characterized by immunohistochemistry. In PBS control EAN rats, infiltration of macrophages (CD68^+^) and T lymphocytes was observed (Figures 3D, 3G). ARA 290 intervention significantly suppressed infiltration of macrophages and T cells (p<0.05) in sciatic nerves (Figures 3E, 3F, 3H, 3I).

In addition, the effects of ARA 290 on the expression of disease-promoting inflammatory cytokines and protective anti-inflammatory cytokines in sciatic nerves of EAN rats were analysed. For Day 14 EAN rats, ARA 290 intervention significantly reduced the mRNA levels IL-1β, IFN-γ and IL-17 (Figure 3J). However, for Day 21 EAN rats, ARA 290 intervention greatly increased the IL-4, IL-10 and and TGF-β level (Figure 3K).

### 3 ARA 290 Enhanced Peripheral Nerves Repair in EAN Rats

**Figure 4:**
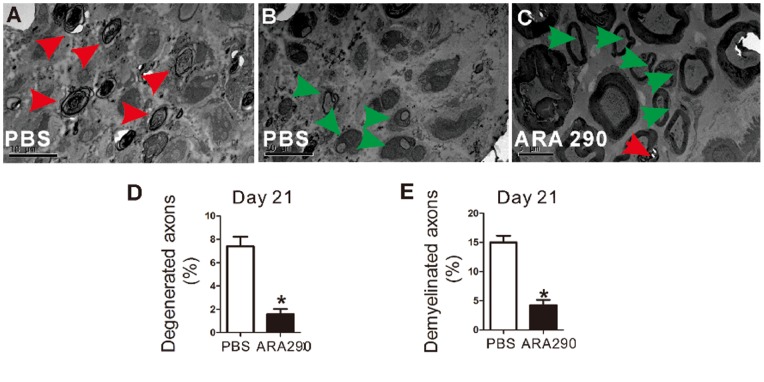
ARA 290 intervention suppressed demyelination and promoted remyelination of sciatic nerves. PBS or ARA 290 (30 mg/kg/day) was given to EAN rats from Day 7 to Day 21 and on Day 21, rats (n = 3) were sacrificed and ultrathin sections of the Day 21 EAN rats sciatic nerves were taken for electron microscopy analysis and representative electron micrographs were shown. **A:** Axon degeneration and demyelination (red arrows) in PBS control EAN sciatic nerves. **B:** Remyelination (green arrows) in PBS control EAN sciatic nerves. **C:** Remyelination (green arrows) and axon degeneration (red arrows) in ARA 290-treated EAN sciatic nerves. *: p<0.05 compared to their respective vehicle control.

EAN is an autoimmune demyelination disorder and axon degeneration can be observed under severe condition. Therefore, axon degeneration/regeneration and demyelination/remyelination following ARA 290 intervention were further investigated in ultrathin sections of the Day 21 EAN rats’ sciatic nerves. Representative electron micrographs depictured pathological changes in EAN nerves. Control nerves with PBS intervention showed more axon degeneration and demyelinated fibres without remyelination (Figure 4). In contrast, in nerves from ARA 290-treated EAN rats more remyelinated fibres and less degenerated axon was observable (Figure 4). Furthermore, morphological studies showed that ARA 290 intervention increased the number of myelinated regenerating axons at sciatic nerve levels in EAN rats compared to PBS-treated EAN rats (Figure 4).

### 4 ARA 290 Suppressed Lymphocyte Proliferation and Altered Helper T cell Differentiation in EAN

**Figure 5:**
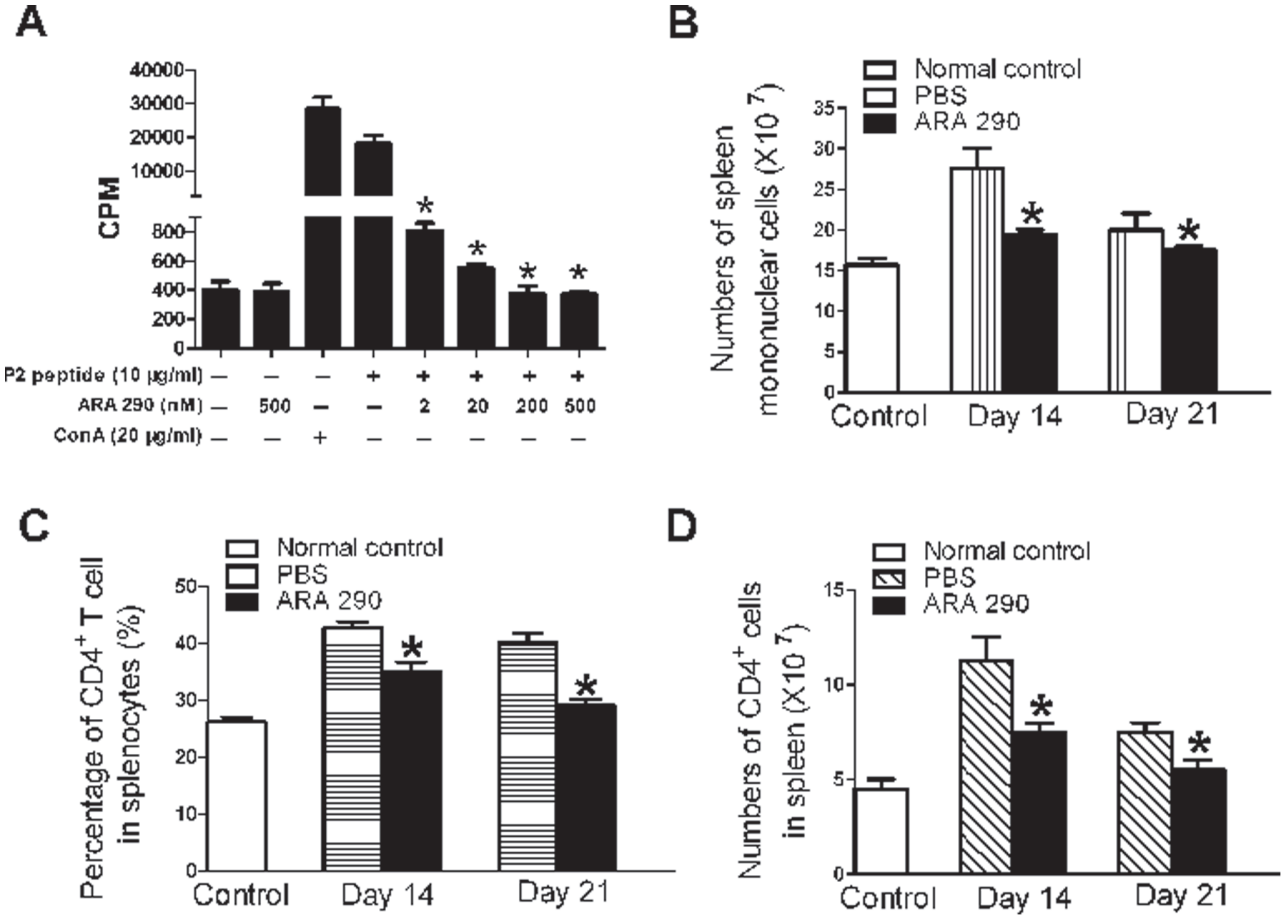
ARA 290 suppressed lymphocyte proliferation *in vitro* and *in vivo*. PBS or ARA 290 was given to EAN rats from Day 7 to Day 21. **A:** In the lymphocyte proliferation assay, spleen mononuclear cells was stimulated by P2 antigen peptide and after a total of 72 hours culture, the specific cell proliferation of spleenocytes by P2 peptide was significantly inhibited by ARA 290. **B:** Numbers of EAN spleen mononuclear cells were counted under a light microscope (n = 3). ARA 290 intervention reduced EAN spleen mononuclear cell numbers on Day 14 and Day 21. **C:** The percentage of CD4^+^ Th cells in EAN spleen was detected by flow cytometric analysis (n = 3). ARA 290 intervention reduced percentage of CD4^+^ Th cells in EAN spleen on Day 14 and Day 21. **D:** The cell number of CD4^+^ Th cells in EAN spleen was calculated. ARA 290 intervention reduced CD4^+^ Th cells in EAN spleen on Day 14 and Day 21. *: p<0.05 compared to their respective vehicle control.

One of the most important immune cells in EAN is the T lymphocyte. The proliferation and polarization of T lymphocytes following auto-antigen stimulation is a central process defining the nature and severity of autoimmune disorders. So we studied effects of ARA 290 on lymphocyte proliferation. As shown in Figure 5A, ARA 290 dose-dependently suppressed antigen specific lymphocyte proliferation in vitro. Furthermore, in EAN rats, the numbers of mononuclear cells and helper T cells (CD4^+^ cells) in spleen were greatly reduced following ARA 290 intervention compared to PBS control (Figure 5B–C), indicating that ARA 290 may inhibite lymphocyte proliferation to contribute to EAN recovery.

**Figure 6:**
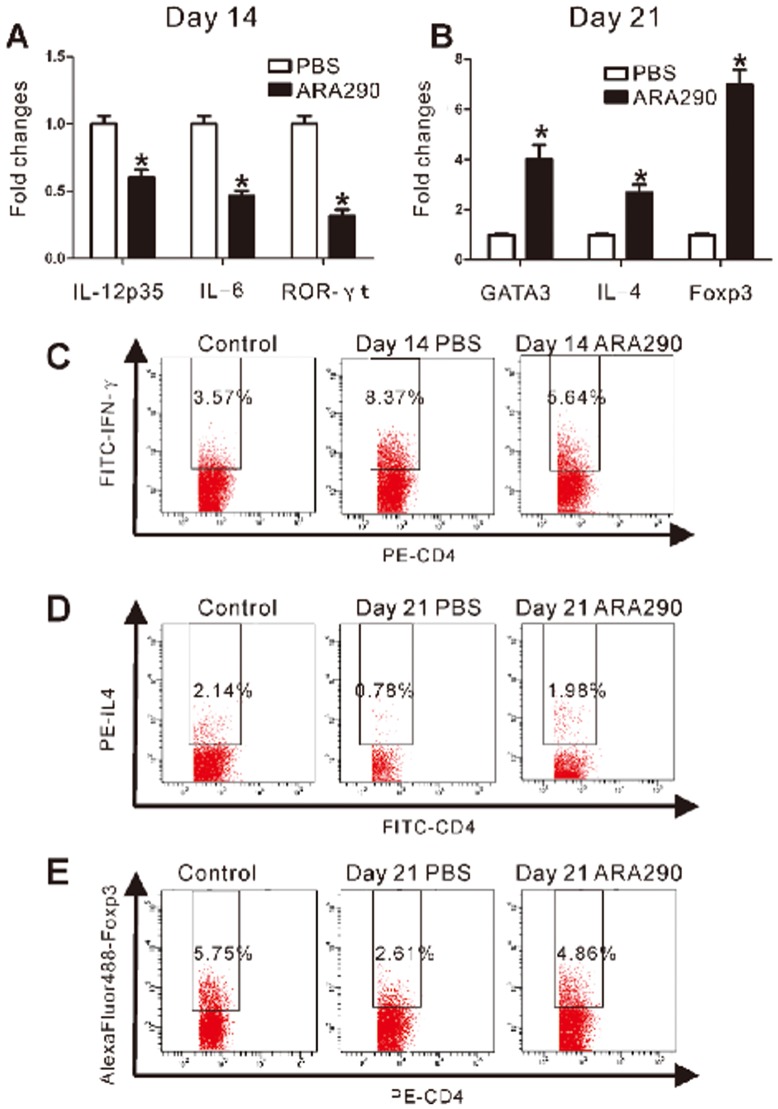
ARA 290 intervention altered Th cell differentiation in EAN. PBS or ARA 290 was given to EAN rats from Day 7 to Day 21 and on Day 14 or Day 21, rats were sacrificed to take inguinal lymph nodes and spleens for RT-PCR or flow cytometric analysis with representative flow cytometry dot plots (n = 3). **A:** In Day 14 EAN inguinal lymph nodes, ARA 290 intervention significantly decreased mRNA levels of IL-12p35, RORγt, and IL-6 compared to the PBS control. **B:** In Day 21 EAN inguinal lymph nodes, ARA 290 intervention significantly increased mRNA levels of GATA3, IL-4 and Foxp3 compared to the PBS group. **C:** In Day 14 EAN spleen, ARA 290 intervention reduced IFN-γ^+^CD4^+^ Th1 cell percentage compared to PBS control. **D:** In Day 21 EAN spleen, IL-4^+^CD4^+^ Th2 cell numbers were increased after ARA 290 intervention compared to PBS control. **E:** In Day 21 EAN spleen, percentage of Foxp3^+^CD4^+^ regulatory T helper cell were increased after ARA 290 intervention compared to PBS control. *: p<0.05 compared to their respective vehicle control.

The draining lymph node is the most important place for helper T cell differentiation so ARA 290 effects on helper T cell differentiation were studied in inguinal lymph nodes of EAN rats. We found that in Day 14 EAN inguinal lymph nodes, ARA 290 intervention significant reduced the mRNA level of RORγt, master transcription factor for Th17 cells, but greatly increased levels of the GATA3, master transcription factor for Th2 cells, and Foxp3, master transcription factor for regulatory T cells, in inguinal lymph nodes of Day 21 EAN rats (Figure 6A). Correspondingly, cytokine IL-12p35 and IL-6, which favors the Th1 and Th17 differentiation, were reduced but cytokine IL-4, implying the Th2 differentiation, was induced (Figure 6A). We further analyzed percentages of Th1, Th2 and regulatory T cells in the spleen at Day 14 and Day 21 EAN rats that were treated by ARA 290. As shown in Figure 6B, ARA 290 intervention significantly reduced the CD4^+^/IFN-γ^+^ Th1 cells in Day 14 EAN spleen but increased the CD4^+^/IL4^+^ and CD4^+^/Foxp3^+^ regulatory T cells in Day 21 EAN spleen compared to the PBS control.

### 5 ARA 290 Inhibited Inflammatory Macrophage Activation but Promoted its Phagocytic Activity *in vitro*

**Figure 7:**
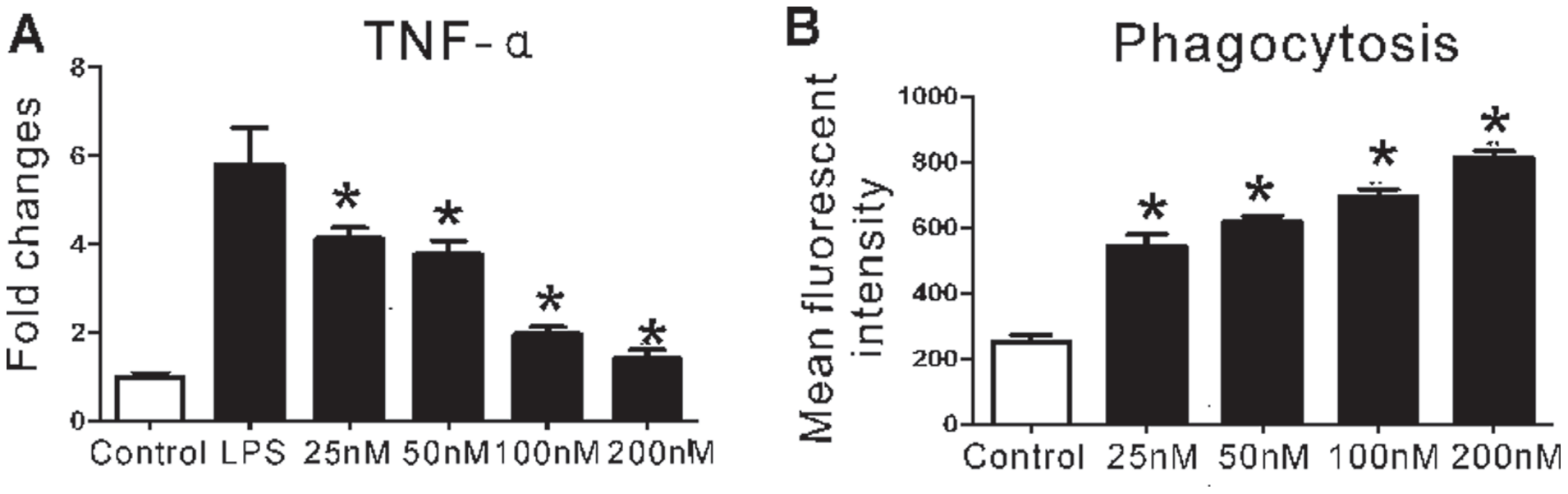
ARA 290 inhibited inflammatory macrophage activation *in vitro*. A: RAW 264.7 cells were treated with 1 µg/mL LPS followed by ARA 290 or PBS control intervention with various doses for 4 hours, TNF-α mRNA level was greatly reduced by ARA 290 intervention. **B:** 0.5 mg/mL FITC-dextran was incubated with RAW264.7 cells for 30 min at 37°C and phagocytosis was measured by flow cytometry. Phagocytosis of FITC-dextran by RAW264.7 cell was greatly up-regulated in presence of ARA 290. *: p<0.05 compared to their respective vehicle control.

In EAN PNS, macrophages have critical functions in promoting inflammation, terminating the immune reaction and promoting peripheral nerve recovery following the autoimmune insult [23]. Therefore here we investigated effects of ARA 290 on macrophages. Our in vitro experiments showed that ARA 290 dose-dependently suppressed LPS-induced inflammatory activation of RAW264.7 macrophage by reducing TNF-α levels (Figure 7A). Interestingly, our results here also showed that ARA 290 greatly increased the phagocytic capacity of macrophages, which is essential to remove apoptotic cells and debris to promote EAN remission, in a dose-dependent way in vitro (Figure 7B).

### 6 ARA 290 Exerted Direct Anti-inflammatory and Proliferation Promotion Effects on Schwann Cells

**Figure 8:**
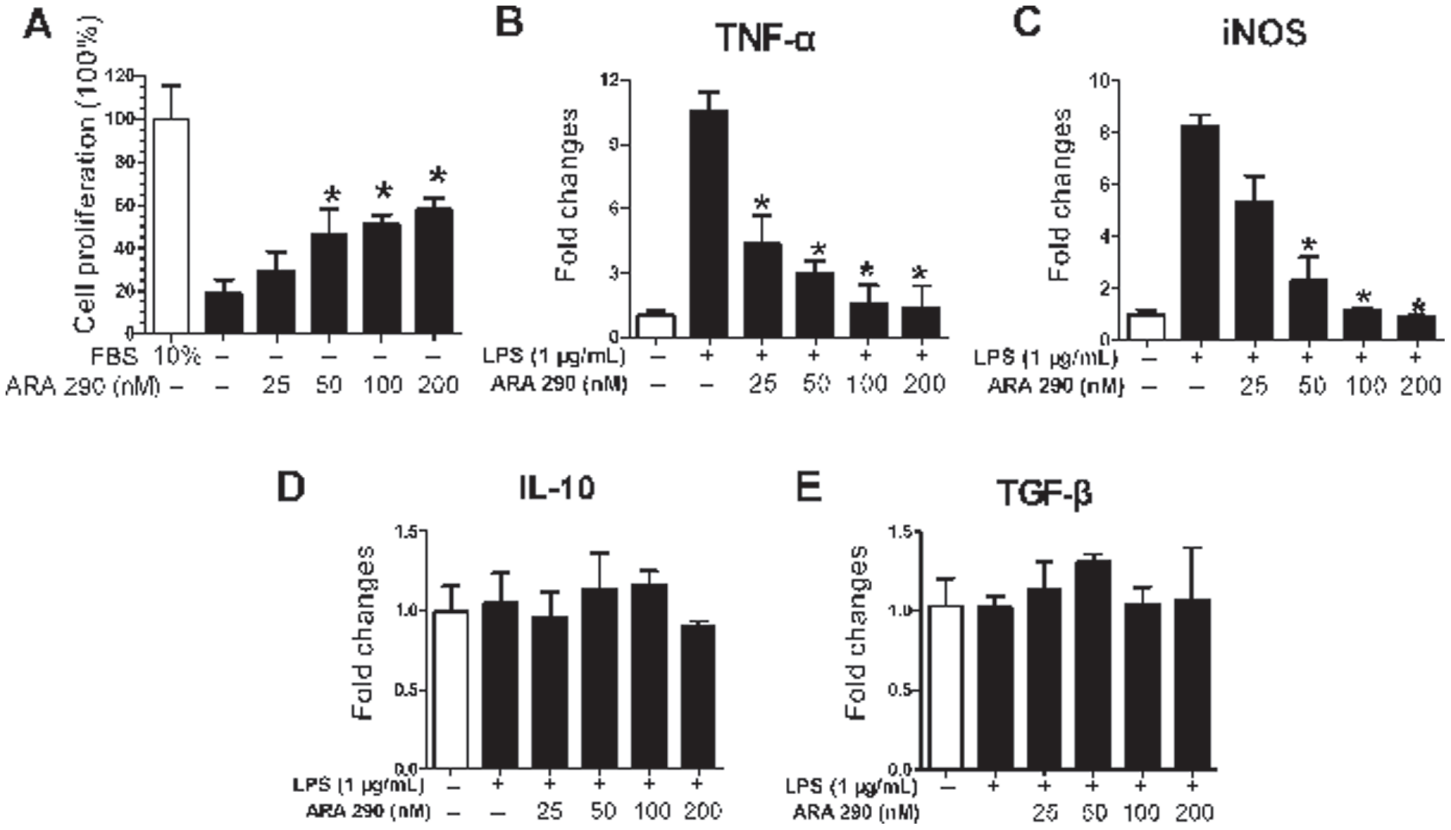
ARA 290 exerted direct proliferation promotion and anti-inflammatory effects on Schwann cells. Effects of ARA 290 on Schwann cells were analyzed in**A:** RSC96 cells were stimulated with ARA 290 for 24 hours without FBS and cell proliferation was detected by CCK-8 analysis. ARA 290 significantly promoted cell proliferation of RSC96 cells. **B–E:** RSC96 cells were stimulated with LPS for 4 hours and then incubated with ARA 290 for 8 hours. mRNA levels of TNF-α and iNOS were significantly reduced by ARA 290, but IL-10 and TGF-β were not observed significant fold changes. *: p<0.05 compared to their respective vehicle control.

Since both endogenous and exogenous ARA 290 was shown to alter demyelination/remyelination in EAN, effects of ARA 290 on rat Schwann RSC96 cells were further analyzed. As shown in Figure 8A, ARA 290 dose-dependently promoted RSC96 cell proliferation in vitro. Furthermore, the effects of ARA 290 on Schwann cell inflammatory activation were analyzed as well. Following LPS stimulation, the mRNA levels of TNF-α and iNOS in RSC96 cells were greatly induced, indicating an inflammatory activation (Figure 8B and C). However, following co-incubation with ARA 290, the up-regulated TNF-α and iNOS was dose-dependently reduced (Figure 8B and C). On the contrary, when co-incubation with ARA 290, anti-inflammatory cytokines TGF-β and IL-10 were not observed significant fold changes (Figure 8D and E).

## Discussion

EAN is the prime animal model for inflammatory demyelinating polyneuropathies and useful in investigating new therapeutic approaches. Here we have studied the effects of ARA 290 on EAN. Our findings demonstrate that ARA 290 greatly reduced paraparesis and peripheral nerve inflammation in EAN rats. ARA 290 intervention inhibited lymphocyte proliferation and favoured the Th2 and Treg differentiation of helper T cells in EAN rats. In addition, ARA 290 inhibited inflammatory macrophage activation and exerted direct cyto-protective and anti-inflammatory effects on Schwann cells. Therefore, our data showed that ARA 290 could be a potent candidate for intervention of autoimmune neuropathies.

While EPO has been shown to improve EAN recovery its erythropoietic activity-induced cardivascular side effects may limit its clinical application. ARA 290 is a short peptide of 11 amino acids, designed for specificity to the EpoR–CD131 heterocomplex and without erythropoietic function [21]. In disease models, such as burn injury, stroke, wound healing, renal ischemia/reperfusion and neuropathic pain, ARA 290 has been reported to promote disease recovery through tissue-protective and immunomodulatory activity [21], [24]–[26]. Our investigation here further proved that ARA 290 has protective effects in autoimmune injury. In addition, accumulated preclinical toxicology studies of ARA 290 have raised no safety issues [27]. Therefore, ARA 290 could be a promising therapeutic candidate for a variety of diseases.

In this investigation, ARA 290 intervention improved EAN outcome and suppressed accumulation of immune cells and inflammatory molecules in peripheral nerves of EAN. Pathological development of EAN is characterized by the infiltration of reactive leukocytes into the PNS [28]. Activated autoreactive helper T cells are of importance for the initiation of EAN [2]. Activated macrophages cause demyelination by direct phagocytic attack and secretion of inflammatory mediators [23] [29], [30]. In peripheral nerves of EAN rats, cytokines are produced and released by many cell types and regulate inflammation and immunity. Pro-inflammatory cytokines, like IFN-γ, IL-1β and IL-17, have disease-promoting roles in EAN and their expression was attenuated by ARA 290. IFN-γ augments both inflammation and subsequent immune responses in EAN by activation of macrophages to release oxygen radicals, promoting T cell and macrophage homing to the PNS, enhancing BNB permeability, inducing MHC-II expression on macrophages and cultured Schwann cells. IL-1β is considered to participate in the initiation of autoimmune response in EAN. IL-17 is produced by Th17 cells and stimulates production of IL-6, nitric oxide and prostaglandin E2 to amplify local inflammation, mediates chemotaxis of neutrophils and monocytes to sites of inflammation and augments the induction of co-stimulatory molecules such as ICAM-1 to support T cell activation [31], [32]. Therefore, ARA 290 attenuated accumulation of inflammatory cells and expression of inflammatory related molecules in PNS could reduce local inflammation and demyelination to favour EAN outcome.

While ARA 290 inhibits inflammation and promotes recovery of EAN the underlying mechanisms remain unclear. EAN is an autoimmune inflammatory demyelinating disorder, and Schwann cells, T cells and macrophages are the major inflammatory and effector cells in EAN, which are essential for the development and recovery of EAN. Our results here clearly showed that ARA 290 could directly influence Schwann cell, macrophage and T cell functions to promote EAN recovery.

T helper (Th) cells are important for the pathogenesis of EAN [2]. Th cell polarization and proliferation following auto-antigen stimulation is essential for the determination of type and severity of autoimmune disorders. In EAN, ARA 290 significantly suppressed lymphocyte proliferation, which may at least partly contribute to the reduced T cell numbers in spleen and therefore disease severity in EAN rats. Furthermore, in EAN, ARA 290 inhibited Th1 and Th17 polarization and promote Th2 and Treg differentiation. EAN is considered to be mainly mediated by Th1 cells and Th1 cytokines, such as IFN-γ or TNF-α [2]. In addition, Th17 cells were believed to contribute to the development of EAN and GBS as well [33]. Th2 cells that may suppress cell-mediated immunity and regulatory T cells that can suppress the activation of the immune system may contribute to the resolution of EAN [34]. Therefore, ARA 290 signal can also alter Th differentiation to favour EAN recovery.

Inflammation-mediated demyelination is the pathogenetic basis of EAN and therefore Schwann cell protection is essential to EAN recovery. In our investigation, ARA 290 enhanced axon remyelination in EAN and in line with this, we observed that ARA 290 dose-dependently increased Schwann cell proliferation in vitro. Therefore, in EAN, ARA 290 has cyto-protective effects on Schwann cell, which are important for the remyelination of the injured nerves.

Macrophages play dual roles in EAN, being detrimental in attacking nervous tissue but also salutary, when aiding in the termination of the inflammatory process and the promotion of recovery [23]. Interestingly, we found that ARA 290 suppressed the inflammatory activation of macrophages and increased the phagocytic ability of macrophages which could be important in the removing of apoptotic cells during the recovery stage of EAN, indicating that ARA 290 can suppress the cytotoxic effects and facilitates the protective effect of macrophages to promote EAN recovery.

In summary, our findings demonstrate that ARA 290 greatly reduced paraparesis and peripheral nerve inflammation in EAN rats. ARA 290 intervention inhibited lymphocyte proliferation and favoured the Th2 and Treg differentiation of helper T cells in EAN rats. In addition, ARA 290 inhibited inflammatory macrophage activation and exerted direct cyto-protective and anti-inflammatory effects on Schwann cells. Therefore, ARA 290 could be a potent candidate for intervention of autoimmune neuropathies.
