# Correction: Erythropoietin-Derived Nonerythropoietic Peptide Ameliorates Experimental Autoimmune Neuritis by Inflammation Suppression and Tissue Protection

**DOI:** 10.1371/journal.pone.0099555

**Published:** 2014-05-30

**Authors:** The PLOS ONE Staff

There are errors in the figure legends for Figures 1 through 4 and in the first sentence of the Results. 30mg should be replaced with 30µg.

**Figure 1:**
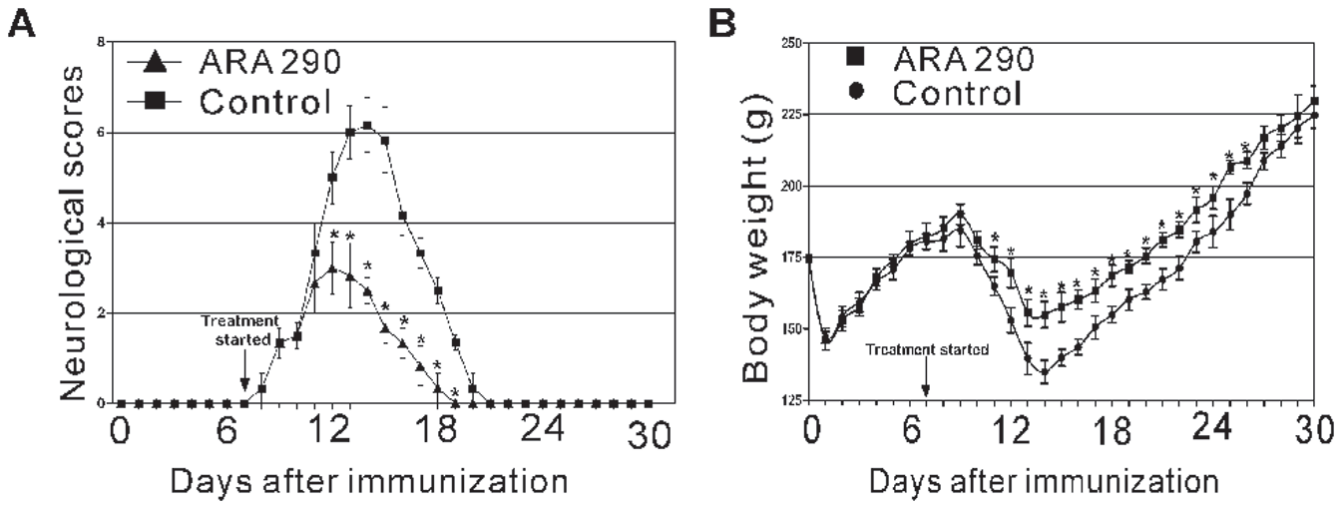
ARA 290 intervention effectively attenuated EAN disease severity. PBS or ARA 290 (30 µg/kg/day) was given to EAN rats (n  =  6) from Day 7 to Day 21. All rats were monitored daily for body weight and neurological signs of EAN. **A:** ARA 290 intervention greatly decreased neurologic severity of EAN, shortened EAN duration in comparison to the PBS-treated group. **B:** ARA 290 intervention reduced body weight loss in comparison to the PBS-treated group. *: p<0.05 compared to their respective vehicle control.

**Figure 2:**
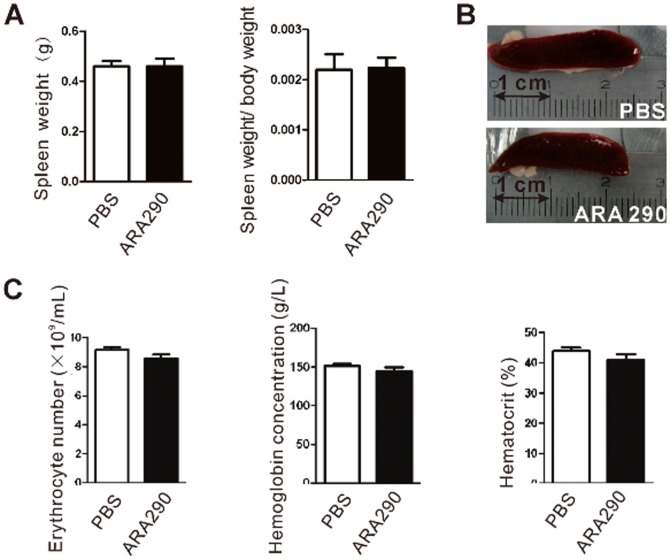
ARA 290 is not erythropoietic *in vivo*. PBS or ARA 290 (30 µg/kg/day) was given to EAN rats (n = 6) from Day 7 to Day 21 and on Day 21, rats were sacrificed to take spleen and pripheral blood for detection of erythropoietic activation. **A–B:** Representative images of spleen was recorded. ARA 290 intervention did not alter the spleen weight of treated rats compared to control. **C:**Anticoagulated blood was measured for erythrocyte cell number, hemoglobin concentration and hematocrit. ARA 290 intervention did not increase erythrocyte cell number, hemoglobin concentration or hematocrit compared to PBS group.

**Figure 3:**
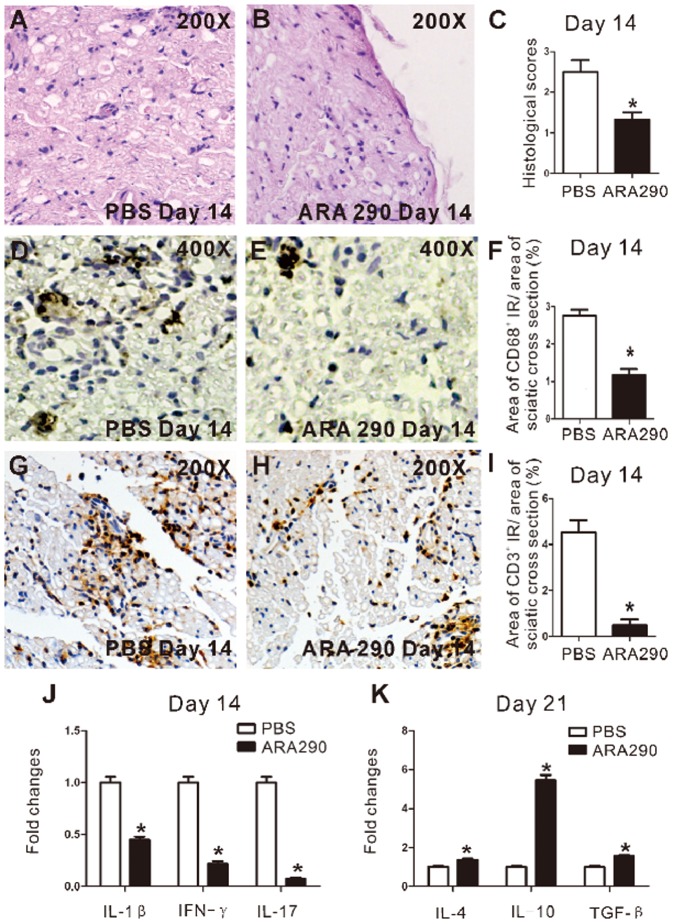
ARA 290 intervention reduced inflammatory cell infiltration and altered inflammatory cytokines expression in sciatic nerves. PBS or ARA 290 (30 µg/kg/day) was given to EAN rats from Day 7 to Day 21 and on Day 14 or Day 21, rats were sacrificed to take sciatic nerves for HE/immunohistochemical staining or RT-PCR analysis. A–C: HE staining was applied to show inflammatory infiltration of Day 14 in EAN sciatic nerves. Representative microimages showed that inflammatory cell infiltration was significantly suppressed by ARA 290 intervention compared to PBS control (n  =  3). D–I: CD68 and CD3 staining was applied to show macrophages and T lymphocytes infiltration of Day 14 in EAN sciatic nerves. Representative microimages showed that macrophages (CD68^+^) and T lymphocytes infiltration was significantly suppressed by ARA 290 intervention compared to PBS control (n  =  3). J–K: Sciatic nerves from Day 14 and Day 21 ARA 290-treated EAN rats were taken for RT-PCR analysis (n  =  3). IL-1β, IFN-γ, IL-17 levels were reduced, while IL-4, IL-10, TGF-βlevels were increased. *: p<0.05 compared to their respective vehicle control.

**Figure 4:**
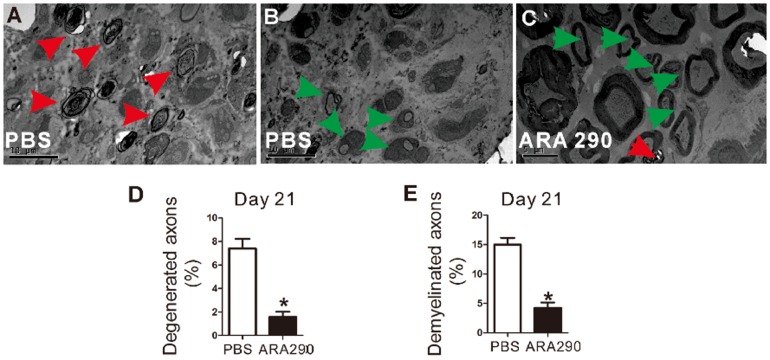
ARA 290 intervention suppressed demyelination and promoted remyelination of sciatic nerves. PBS or ARA 290 (30 µg/kg/day) was given to EAN rats from Day 7 to Day 21 and on Day 21, rats (n = 3) were sacrificed and ultrathin sections of the Day 21 EAN rats sciatic nerves were taken for electron microscopy analysis and representative electron micrographs were shown. **A:** Axon degeneration and demyelination (red arrows) in PBS control EAN sciatic nerves. **B:** Remyelination (green arrows) in PBS control EAN sciatic nerves. **C:** Remyelination (green arrows) and axon degeneration (red arrows) in ARA 290-treated EAN sciatic nerves. *: p<0.05 compared to their respective vehicle control.

The first sentence of the Results should read: “EAN rats administrated ARA 290 (30 µg/kg/day) or PBS (control group) from Day 7 to 21.”

Figures 1 through 4 and their corrected figure legends are provided here.
